# Terahertz Antenna-Coupled Wire-Channel Field-Effect Transistors Based on AlGaN/GaN Heterostructures

**DOI:** 10.3390/s26092701

**Published:** 2026-04-27

**Authors:** Maxim Moscotin, Justinas Jorudas, Pawel Prystawko, Miroslav Saniuk, Vitalij Kovalevskij, Irmantas Kašalynas

**Affiliations:** 1 Terahertz Photonics Laboratory, Center for Physical Sciences and Technology (FTMC), LT-10257 Vilnius, Lithuania; justinas.jorudas@ftmc.lt (J.J.); miroslav.saniuk@ftmc.lt (M.S.); vitalij.kovalevskij@ftmc.lt (V.K.); 2 Laboratory of Semiconductor Characterization, Institute of High Pressure Physics PAS (UNIPRESS), 01-424 Warsaw, Poland; pprysta@unipress.waw.pl

**Keywords:** terahertz detection, AlGaN/GaN HEMT, antenna-coupled FET, modified EdgeFET, plasma-wave rectification, TeraFET, GaN terahertz devices

## Abstract

We propose a terahertz (THz) antenna-coupled wire-channel field-effect transistor—modified EdgeFET (m-EdgeFET), formed by combining single-gate FinFET and dual-side-gate EdgeFET concepts, which is used for THz detection. The proposed hybrid design was implemented on AlGaN/GaN high-electron-mobility transistor (HEMT) structures, demonstrating distinct response characteristics under 150 GHz and 300 GHz radiation at room temperature. The responsivity dependence on the channel length was determined, revealing that the peak responsivity reached up to 6.5 V/W at a gate voltage of −3 V, i.e., at a gate bias that is an order lower in magnitude than that required for EdgeFET to reach the maximum response. Meanwhile, the gate leakage current decreased by an order of magnitude (to about 1 nA) compared to a FinFET with similar geometry. The proposed geometry was shown to operate in two regimes: source-drain coupling (SD) and gate coupling (GG) of THz radiation with the transistor wire channel. The results confirm that the m-EdgeFET design is suitable for electrically controlled and fast THz detection.

## 1. Introduction

Despite broad potential, the widespread adoption of THz technology remains limited by the lack of fast, compact, and cost-effective THz detectors capable of operating at room temperature. Conventional THz detectors such as Golay cells and bolometers suffer from slow (millisecond) response times [1,2]. Antenna-coupled microbolometers, recognized as sensitive room-temperature THz detectors, are available for room-temperature THz imaging and spectroscopy, possessing sensitivity values in the range of 1000 V/W with a typical response time below 1 μs [3,4]. On the other hand, photoconductive antennas, widely used in laboratory THz time-domain spectroscopy systems, deliver sufficient temporal resolution but rely on femtosecond laser excitation, which limits their applicability in stand-alone or integrated platforms [5]. These constraints underscore the need for solid-state detectors that combine room-temperature operation, fast response, compactness, and electrical tunability. In practice, THz detector development is often a trade-off between spectrally selective mechanisms and broadband room-temperature operation. One promising pathway is provided by field-effect transistors, which, coupled to a THz antenna, act as terahertz detectors (TeraFETs) [6] owing to their intrinsic nonlinearity. The theoretical foundation for this concept originates from the Dyakonov–Shur model [7], which predicted that two-dimensional plasma waves can be excited within the channel of a transistor under THz irradiation [8]. Depending on the carrier scattering time and channel geometry, plasma oscillations can operate in two distinct regimes. In the resonant regime, standing plasma waves can form between the source and drain boundaries, leading to resonance features at frequencies determined by the channel length and electron density [9]. This regime offers high spectral selectivity but typically requires reduced damping and often cryogenic operation [8]. In the overdamped (non-resonant) regime, plasma oscillations decay rapidly, yet the nonlinearity of the transistor channel allows rectification of the THz signal into a measurable DC voltage or current [10,11]. This mechanism enables broadband, room-temperature operation, which is highly attractive for practical detector applications. In both regimes, the transistor serves a dual function: it acts as a plasmonic cavity where THz fields couple into the channel and as a nonlinear rectifier that converts high-frequency oscillations into a convenient direct output signal [11,12].

AlGaN/GaN high-electron-mobility-transistor (HEMT) structures have emerged as a particularly promising material platform for TeraFETs [13]. While GaN-based devices do not exhibit superior electron mobility compared to arsenide-based HEMTs, their high electron saturation velocity and pronounced nonlinear transport enable efficient THz rectification in both resonant and overdamped regimes [14]. In addition, the high breakdown field and thermal robustness of AlGaN/GaN heterostructures make them attractive for sensing applications in demanding environments [11,15]. Recent progress in room-temperature TeraFETs has demonstrated that antenna-coupled field-effect transistors can operate over a broad frequency range and can be engineered for improved sensitivity through antenna design, channel scaling, and device-layout optimization [6,11,13,14]. In particular, recent monolithically integrated AlGaN/GaN HEMT detectors have reported optical sensitivities exceeding 20 mA/W up to 1 THz and best optical noise-equivalent power values below $10^{-11}\;W/\sqrt{\mathrm{Hz}}$ at 175 GHz, with NEP values remaining below $10^{-10}\;W/\sqrt{\mathrm{Hz}}$ over broad spectrum ranges reaching frequencies up to 900 GHz [16]. At the same time, among room-temperature THz direct detectors, highly optimized zero-bias Schottky diodes remain an important broadband sensitivity benchmark, with reported NEP values of about $10^{-11}\;W/\sqrt{\mathrm{Hz}}$ in the range of 0.2–1.2 THz [17].

Despite significant progress in AlGaN/GaN TeraFETs, important challenges remain: the responsivity can still be limited by competing parasitic oblique plasma modes, by carrier scattering at device edges, and by inefficient coupling between the antenna and the channel [18]. To overcome these problems, wire-channel transistor architectures such as EdgeFETs were introduced [19,20]. In these devices, two laterally separated gate electrodes approach the channel from opposite sides, and by restricting gating to the channel edges, this geometry suppresses the formation of oblique plasma modes. It promotes predominantly longitudinal plasma oscillations, improving coupling efficiency and enhancing the detector response.

In this work, we introduce a modified EdgeFET (m-EdgeFET) concept for room-temperature THz detection that combines elements of the edge-shaped gate (EdgeFET) and fin-shaped gate (FinFET) 2DEG transistor architectures. The motivation for this hybrid design is not only improved gate control. The side-gating electrodes inherited from the EdgeFET geometry help preserve edge-defined excitation and suppress parasitic oblique modes, while the narrow interconnection between the split gates provides additional top-gate control, lowers the magnitude of the depletion voltage, and increases the effective gate–channel capacitance. As a result, m-EdgeFET is expected to combine the low-voltage operation of a top-gated device with the field localization advantages of an EdgeFET. We show that this geometry enables electrical control of both the effective channel width and the active gated length, which is beneficial for room-temperature THz detection and for comparing source–drain and gate–gate coupling regimes.

## 2. Materials and Methods

**Figure 1:**
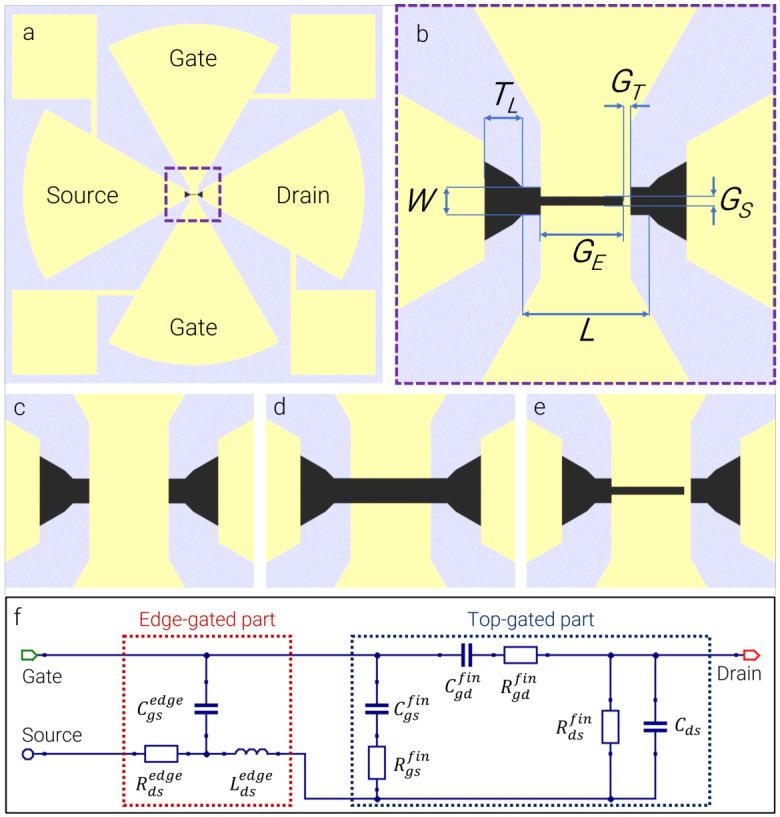
(**a**) TeraFET design overview with two perpendicular bow-tie antennas (yellow), dimensioned for operation around 300 GHz: arms of one antenna are connected to the channel (black), and arms of the other are connected to the gate(s); (**b**) zoom-in of the TeraFET channel part with dimensions from Table 1 indicated; channel overview of (**c**) FinFET—one solid gate electrode; (**d**) EdgeFET—two gates separated by 3 μm; (**e**) m-EdgeFET—two parts of the gate separated by 1 μm and locally connected by a narrow bridge. (**f**) Internal lumped-element scheme of the m-EdgeFET device proposing usage of a resistive self-mixing in the top-gated channel and LC-coupling in the edge-gated channel regions for THz detection.

A series of devices with systematically varied channel lengths and gate configurations (Figure 1) was designed to investigate the THz detection efficiency of the three gate concepts. The design parameters of all samples are summarized in Table 1. The channel width was fixed at 3 μm, while the channel length was set to 9 μm, 13.5 μm, and 21 μm. These values were chosen to probe channel-length scaling while keeping a practically achievable large aspect ratio between the width and length of the wire-channels to allow direct comparison between FinFET, EdgeFET, and m-EdgeFET layouts within the same fabrication run. Three gate layouts were realized: (i) a FinFET configuration (labeled c1), featuring a continuous gate electrode covering a large part of the channel (Figure 1c); (ii) an EdgeFET configuration (labeled d1, d2, d3), consisting of two laterally separated gate electrodes with 3 μm spacing approaching the channel from opposite sides (Figure 1d); and (iii) an m-EdgeFET configuration (labeled e1, e2, e3), with the opposing gate electrodes partially covering the channel from both sides, separated by 1 μm and connected locally by a narrow bridge asymmetrically placed over the wire channel (Figure 1e). In the DC regime, this bridge acts as a short top gate and reduces the depletion voltage required for channel closure compared with a pure EdgeFET. Under THz excitation, the same modification alters the local capacitance and field distribution relative to both the EdgeFET and FinFET geometries. The narrow spacing between the gate electrodes also introduces an internal channel inductive contribution whose plasmonic and kinetic behavior varies with applied bias [12,21].

To clarify the antenna–device coupling and frequency behavior, we introduce a minimal small-signal lumped-element representation of the devices (Figure 1f). In this model, the effective device impedance under THz excitation is governed by the channel resistance $R_{\mathrm{ch}}\left(V_{G}\right)$ and the gate capacitance $C_{G}\left(V_{G}\right)$. The representation allows distinguishing between source–drain (SD) and gate–gate (GG) excitation schemes and illustrates how the modified gate geometry of the m-EdgeFET alters the capacitive coupling paths and effective RC time constant. In particular, the additional inter-gate bridge increases the effective gate–channel capacitance while simultaneously reducing the required depletion voltage, thereby modifying the high-frequency response compared to the EdgeFET and FinFET configurations. Section S1 of the Supplementary Material presents simulated frequency-dependent impedance characteristics of the bow-tie antenna.

The detector structures were fabricated from AlGaN/GaN HEMT heterostructures grown on a 4-inch, 500 μm thick semi-insulating SiC substrate. The heterostructure consisted of a 2.4 nm GaN cap, a 20.5 nm Al_0.25_Ga_0.75_N barrier, and a 255 nm GaN channel grown directly on a 62 nm high-quality AlN nucleation layer on SiC. The as-grown sheet resistance determined by Hall-effect measurements was approximately 375Ω/sq, while the two-dimensional electron gas density was of the order of $1\times 10^{13}\;{\mathrm{cm}}^{-2}$. The detector devices were defined using standard ultraviolet photolithography. Mesa isolation was formed by inductively coupled plasma reactive ion etching using Cl plasma to a depth of approximately 140 nm, followed by chemical treatment in tetramethylammonium hydroxide solution. Ohmic contacts were formed from Ti/Al/Ni/Au metal stacks with thicknesses of 30/90/20/150 nm and annealed in nitrogen ambient at 850 °C for 30 s. Schottky gate contacts with a 0.75 eV barrier and a low-resistance connection to the gate antenna arms were formed from Ni/Au with thicknesses of 25/150 nm. All devices, FinFET, EdgeFET, and modified EdgeFET detector layouts were fabricated together with the corresponding bow-tie antennas, enabling a direct comparison of the effect of gate geometry on the DC transistor characteristics and THz response. More details about the HEMT structures used, in-house processing of the electric contacts, and research on voltage-current characteristics have been published elsewhere [15].

For the normally-on devices studied here, the threshold voltage $V_{\mathrm{th}}$ is defined operationally as the gate voltage corresponding to channel closure in the measured transfer characteristics. In practice, this value was identified from the $I_{D}$–$V_{G}$ curves as the bias at which the derivative $dI_{D}/dV_{G}$ changes sign, marking the transition from a conducting channel to an effectively depleted state. Thus, in the present work, $V_{\mathrm{th}}$ should be understood as a practical channel-closing or pinch-off voltage rather than as a universal threshold voltage extracted from an ideal long-channel MOSFET model. This distinction is important because the investigated device geometries differ substantially in gate layout, depletion profile, and field distribution, so the corresponding threshold values are geometry-dependent and should be compared only in this operational sense.

**Figure 2:**
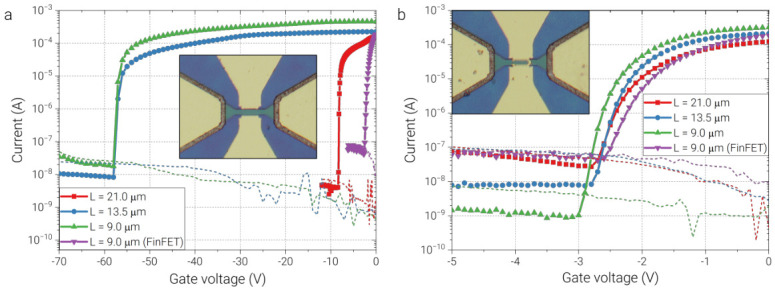
Solid-dotted lines: transfer characteristics ($I_{D}$–$V_{G}$) of TeraFETs with channel lengths of 21 μm, 13.5 μm, and 9 μm in (**a**) EdgeFET and (**b**) m-EdgeFET configurations, measured at $V_{\mathrm{DS}}=1\;V$. A 9 μm FinFET device is included in both panels for reference. Dashed lines: gate leakage currents ($I_{G}$–$V_{G}$).

To extract $V_{\mathrm{th}}$, transfer ($I_{D}$–$V_{G}$) characteristics were measured for the fabricated samples on a probe station at a fixed drain–source bias of 1 V. The results are shown in Figure 2. The EdgeFET samples (Figure 2a) exhibited comparatively large-magnitude closing voltages, ranging from about −8 V to approximately −80 V, with the smallest magnitude observed for the longest wire channel. This behavior is consistent with the purely lateral edge-gating geometry, where channel depletion is induced mainly from the sides and therefore requires substantially stronger negative bias to fully close the conducting path. In contrast, the m-EdgeFET and FinFET structures exhibited much smaller threshold-voltage magnitudes, within the range of about −2.5 V to −3 V (Figure 2b), because the added top-gate component provides much stronger electrostatic control over the channel. In the m-EdgeFET, the narrow bridge between the split gates acts as a short top-gated region, reducing the voltage required for channel depletion compared with the pure EdgeFET while preserving the edge-defined geometry.

It is also important to compare the closed-state leakage currents, because a low threshold voltage alone does not guarantee better detector operation. The $I_{G}$–$V_{G}$ characteristics shown in Figure 2 (dashed lines) indicate that for the 9 μm channel-length devices, m-EdgeFET retains a particularly low closed-state current of about 1 nA at $V_{G}=-3$ V. Under comparable closing conditions, 9 μm EdgeFET exhibits a closed-state current of about 19.5 nA at $V_{G}=-60$ V, while 9 μm FinFET reaches about 52.5 nA at $V_{G}=-3$ V. Therefore, m-EdgeFET combines the advantage of low-voltage channel closure with lower leakage in the closed state than the corresponding FinFET while requiring far smaller closing voltages than the EdgeFET. This comparison further supports the view that the modified EdgeFET geometry provides a more favorable balance between electrostatic controllability and practical operating bias for THz detector applications.

## 3. Results and Discussion

Responsivity measurements were performed in two assemblies: with and without a silicon (Si) hyperhemispherical focusing lens attached to the rear side of the chip to enable back-side illumination. The samples were mounted in a cage system that allowed us to switch between two cases, broad-beam measurements and tightly focused-beam measurements, by attaching or removing the Si lens while maintaining repeatable positioning. The chip–lens assembly was mounted on an XYZ translation stage for precise alignment of the selected sample. Measurements were carried out using linearly polarized radiation of two frequencies, 150 GHz and 300 GHz, sourced from a diode multiplier chain and modulated at 1 kHz. During operation, the source electrode was grounded, and the drain was connected to the input of a lock-in amplifier. The response voltage as a function of the sample position was recorded in the focal plane of the off-axis parabolic (OAP) mirror. First, the beam profiles without bias on the TeraFET were obtained. Then, the detected signal dependence on the gate bias $V_{G}$ was measured while keeping the sample in the focal spot, which resulted in peak responses near the transistor threshold voltage. The optical responsivity [22] was calculated as the ratio of the measured response voltage to the total power of the incident THz beam, measured with a calibrated power meter (Thomas Keating). The THz response of each TeraFET sample was measured in two distinct regimes: channel-coupled (SD), when the antenna connected to the source–drain terminals is coupled with the incident radiation, and gate-coupled (GG), when the antenna connected to the gate terminals is coupled with the radiation. In both regimes, the measured responsivity as a function of gate voltage reproduced the functional dependence of the transconductance, obtained from the slope of the measured transfer characteristics ($g_{m}=\partial I_{D}/\partial V_{G}$). The results confirm that the detection originates from the nonlinear dependence of the channel conductance on gate bias due to plasma-wave rectification [23,24].

The THz response of the investigated devices is interpreted within the overdamped plasma-wave self-mixing framework. In this regime, the incident THz field induces alternating carrier-density and drift-velocity perturbations in the transistor channel, while the intrinsic nonlinearity of the channel conductivity leads to rectification into a measurable DC output signal. For a FET operating in the non-resonant regime, the voltage responsivity is expected to scale with the gate-voltage dependence of the channel conductivity, and may be written in a simplified form as $R_{v}\propto \left(1/\sigma \right)\;\left(\partial \sigma /\partial V_{G}\right)$, or equivalently through the transconductance-related nonlinearity of the transfer characteristics. Therefore, the strongest detector response is expected in the bias range where the conductivity changes most rapidly with gate voltage, i.e., near the onset of channel depletion rather than deep in the open-channel regime or after full channel closure.

The distinction between the source–drain (SD) and gate–gate (GG) coupling schemes can be understood within the same small-signal picture. In the SD configuration, the incident THz field is coupled predominantly between the source and drain terminals, producing an AC modulation of the channel potential and a longitudinal electric field along the conducting path. In the GG configuration, the THz field is coupled predominantly to the gate system, leading to stronger direct modulation of the depletion region and gate-controlled capacitance. As a result, the two schemes probe different field distributions and different effective nonlinearities of the same transistor. Their relative efficiency, therefore, depends not only on the external antenna orientation but also on the bias-dependent channel resistance, gate capacitance, and localization of the AC potential drop in the active part of the channel.

**Figure 3:**
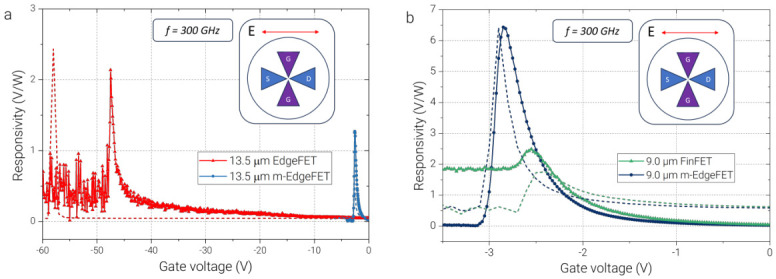
Comparison of the gate-bias-dependent voltage responsivity of TeraFETs with different gate geometries at 300 GHz under source–drain (SD) coupling. (**a**) Comparison of the 13.5 μm EdgeFET and 13.5 μm m-EdgeFET devices. (**b**) Comparison of the 9.0 μm FinFET and 9.0 μm m-EdgeFET devices. The solid curves show the measured THz responsivity as a function of gate voltage, while the dashed curves show the corresponding transconductance $g_{m}$ (in arbitrary units) derived from the transfer characteristics and plotted for comparison with the detector response. In both panels, the responsivity maxima occur close to the gate-bias region where the transconductance is largest, supporting the interpretation of the detector signal in terms of overdamped plasma-wave self-mixing. The insets schematically indicate the orientation of the incident electric field with respect to the antenna and terminal configuration used for SD coupling.

Depending on the gate geometry, a different THz response dependence on gate voltage is observed. Figure 3 compares the 300 GHz responsivity of several TeraFET configurations with differing gate layouts. In Figure 3a, both the 13.5 μm EdgeFET and 13.5 μm m-EdgeFET devices demonstrate pronounced peak responsivities exceeding 1 V/W in the vicinity of the threshold voltage. However, EdgeFET closes at much higher negative gate voltages, indicating a lateral gating effect and therefore a higher effective threshold. We note that the transconductance of the 13.5 μm EdgeFET sample was measured directly by using two source meters sharing a common ground, whereas the THz response was acquired using isolated grounds for a source-measure unit and a lock-in amplifier. Such differences in measurement setups caused slight displacement between the gate-bias positions of the transconductance peak and the corresponding peak in THz responsivity for the EdgeFET samples. In m-EdgeFET, the narrow bridge connecting the opposing gate sections modifies both the electrostatics and the high-frequency coupling. In the DC regime, it acts as a short top-gated segment and reduces the magnitude of the voltage required to deplete the channel compared with a pure EdgeFET. Under THz excitation, the same feature increases the effective gate–channel capacitance and redistributes the local AC field in the vicinity of the wire channel. Consequently, m-EdgeFET can reach a strong nonlinear response at lower gate bias than EdgeFET while still preserving the edge-defined field localization absent in a purely continuous FinFET gate.

In Figure 3b, 9 μm FinFET and 9 μm m-EdgeFET both demonstrate a low-voltage threshold. FinFET exhibits a smaller 2.5 V/W peak and a constant non-zero response beyond the threshold due to the absence of full transistor closure. In contrast, m-EdgeFET shows a pronounced peak exceeding 6 V/W. This behavior can be explained by the reduced gate–channel overlap area in the m-EdgeFET geometry, which minimizes gate leakage and results in a sharper peak at the threshold gate voltage with minimum response in the closed state of the transistor. The improved responsivity of the m-EdgeFET is directly reflected in its noise-equivalent power (NEP). The minimum NEP at 300 GHz reaches $2.6\times 10^{-8}$ W/$\sqrt{\mathrm{Hz}}$ for 9 μm m-EdgeFET, whereas the corresponding 9 μm FinFET exhibits a minimum NEP of $6.4\times 10^{-8}$ W/$\sqrt{\mathrm{Hz}}$ under identical operating conditions. The NEP was estimated from the measured voltage responsivity and the Johnson–Nyquist thermal noise of the device resistance. In this approach, the noise spectral density is taken as $V_{n}=\sqrt{4k_{B}TR}$, where $k_{B}$ is the Boltzmann constant, *T* is the temperature, and *R* is the measured channel resistance at the corresponding bias point. The NEP is then calculated as $\mathrm{NEP}=V_{n}/R_{v}$, where $R_{v}$ is the measured voltage responsivity. Thus, the reported values correspond to an optical NEP referenced to the incident THz power at the detector position. Other possible contributions, such as shot noise and 1/f noise, were not included in this first-order estimate and are expected to become more important only in specific bias and frequency ranges. A comparison of responsivity and NEP values for different gate geometries is shown in Table 2. More details about NEP are provided in Section S3 of the Supplementary Material. We note that these values are obtained from the responsivity referenced to the incident THz power at the detector position, without normalization to the absorbed power and effective area of the used bow-tie antenna. For the EdgeFET device, the NEP was not evaluated because the DC transport characteristics and the THz response were measured in different experimental configurations, preventing consistent extraction of the channel resistance under the same gate bias required for the NEP calculation.

**Figure 4:**
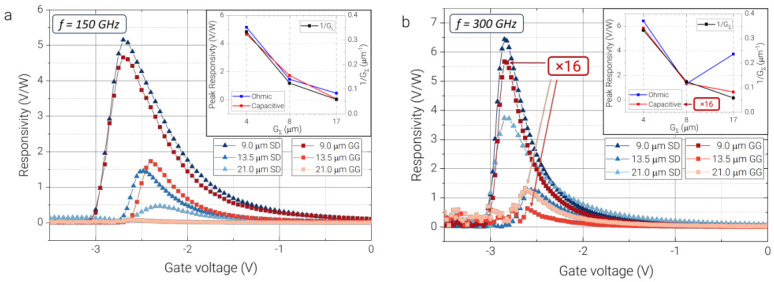
Gate-bias-dependent THz voltage responsivity of m-EdgeFET detectors with three channel lengths measured in both source–drain (SD) and gate–gate (GG) coupling configurations: (**a**) at 150 GHz and (**b**) at 300 GHz. The channel lengths are L=9.0, 13.5, and 21.0 μm. Solid symbol/line sets correspond to the measured responsivity as a function of gate voltage for the different devices and coupling schemes. For both frequencies, the shortest device exhibits the highest peak responsivity, while the response generally decreases with increasing channel length. The insets summarize the peak responsivity as a function of effective gated length $G_{\Sigma }$ for the two coupling schemes and compare the experimental trend with the geometric scaling $1/G_{\Sigma }$, shown for reference.

A set of m-EdgeFET devices with different channel lengths (21 μm, 13.5 μm, and 9 μm) was measured at two selected frequencies of 150 GHz and 300 GHz. The results obtained at 150 GHz are shown in (Figure 4a). The measurements demonstrated an inverse dependence of responsivity on the channel length; hence, shorter-channel devices provide higher responsivity due to more efficient nonlinear self-mixing. The decrease in responsivity with increasing channel length can be attributed to the larger ballast resistance of the channel, which does not contribute to the plasma-wave rectification [24]. Clear differences between the devices appear: the shortest-channel transistors show the largest peak responsivity, while the amplitude decreases systematically with increasing *L* for both coupling geometries. This behavior is quantified in the inset, where the extracted peak responsivity is plotted against the length of the total gated channel part ($G_{\Sigma }=G_{E}+G_{T}$) and compared with $1/G_{\Sigma }$. Both SD and GG coupling data follow this approximate $1/G_{\Sigma }$ dependence over three length values. This monotonic decrease with channel length is consistent with operation in the overdamped self-mixing regime, where ωτ≪1 (ω is the angular frequency, τ is the relaxation time) and the intrinsic plasma-wave decay length is much smaller than any of the fabricated channels. Consequently, only a small portion of the channel close to the source participates in the rectification process, while the remaining segment acts primarily as series (ballast) resistance. Increasing $G_{\Sigma }$ therefore reduces the detected voltage even when the intrinsic self-mixing factor $\left(1/\sigma \right)\left(\partial \sigma /\partial V_{G}\right)$ remains effectively geometry-independent [24]. At 300 GHz (Figure 4b), the channel-length dependence remains present but becomes less systematic and more sensitive to the coupling configuration. The shortest-channel devices still produce the highest responsivity, yet the relative reduction with $G_{\Sigma }$ is weaker than at 150 GHz. The inset reveals that the length dependence deviates from the simple $1/G_{\Sigma }$ scaling and that the two coupling geometries no longer follow the same trend.

Although ωτ increases at 300 GHz compared to 150 GHz, the devices remain in the overdamped regime. Using a conservative value of τ≈0.3 ps for AlGaN/GaN heterostructures extracted from THz spectroscopy measurements [25,26], we obtain ωτ≈0.28 at 150 GHz and ωτ≈0.57 at 300 GHz. Thus, while the excitation approaches the intermediate regime, ωτ still remains below unity, and the channel length is significantly larger than the plasma decay length, reaching approximately 0.4 μm for 300 GHz. Therefore, the detection mechanism is governed by overdamped plasma-wave self-mixing rather than by transit-time–limited propagation across the full channel. The relevant length scale is the plasma decay length ℓ(ω), which decreases with increasing frequency, and confines the effective mixing region closer to the excitation contact. As a consequence, at 300 GHz, the rectification becomes more localized near the injection region, and the spatial distribution of the high-frequency field—determined by the specific gate geometry and coupling configuration—plays a more pronounced role. Overall, the results at 150 GHz and 300 GHz demonstrate that the THz responsivity of long-channel GaN TeraFETs is governed by the interplay between resistive self-mixing in the top-gated channel and LC-mediated signal mixing in the edge-gated regions. This behavior indicates that the device response can be described by a lumped-element model incorporating a series resistance and a bias-dependent kinetic inductance $L_{G}$, both determined by the spatial distribution of the electric field within the channel. At lower frequencies, where plasma waves are strongly overdamped, the response follows a simple $1/G_{\Sigma }$ trend. At higher frequencies, deviations from this scaling emerge as the excitation increasingly probes the spatial field distribution and inter-$L_{G}$–$C_{G}$ element coupling in the edge-gated channel, while still remaining within the overdamped transport regime. Although the present experimental study is limited to 150 and 300 GHz, the proposed geometry is, in principle, scalable to higher frequencies. In that case, the role of parasitic capacitances, access resistances, and field localization becomes increasingly important, while the characteristic plasma-decay-length decreases further. The m-EdgeFET concept remains attractive because the added bridge improves electrostatic control and lowers the operating depletion voltage; however, for operation toward the sub-THz and THz range above 1 THz, the bridge capacitance and antenna–device impedance matching would require more thorough optimization.

To demonstrate the fast response speed of the TeraFET samples, the incident THz radiation was modulated up to 1 MHz, and the detected signal was recorded directly using an oscilloscope with an input impedance of 1 MΩ. At zero gate bias, the signal of the m-EdgeFET sample with 9 μm long wire-channel exhibited rise/fall times of 3 μs and 0.7 μs, respectively (maximum modulation of 270 kHz). At the gate bias of the peak of transconductance (−2.5 V for this m-EdgeFET), the detector signal exhibited rise/fall times of 8 μs and 7 μs, respectively (maximum modulation of 66 kHz). The response time of the detector follows the characteristic RC time constant, with larger channel resistance or capacitance leading to longer rise and fall times and lower modulation bandwidth. The measured response-speed characteristics should be interpreted as the bandwidth of the detector together with the readout and measurement circuitry. Therefore, the observed cut-off frequencies and rise/fall times represent the performance of the present experimental implementation and should not be interpreted as an intrinsic limitation of the detector itself. The slower response under applied gate bias is consistent with an increased effective RC time constant of the biased device and measurement circuit.

## 4. Conclusions

In summary, the modified EdgeFET (m-EdgeFET) concept has been proposed, enabling simultaneous electric control of both the charge-carrier density in the two-dimensional electron gas and the effective width and length of the wire-channel transistor. This design overcomes limitations of other geometries: larger gate leakage in FinFETs and higher negative threshold voltages in EdgeFETs. The developed m-EdgeFETs demonstrate improved electrostatic control, achieving lower threshold voltages and reduced gate-leakage currents while maintaining equal or higher THz responsivity compared with conventional gate configurations. The experimental results show that m-EdgeFET devices can perform broadband THz detection at room temperature, operating at a practical gate bias of −3 V, with responsivity values up to 6.5 V/W. These findings confirm that combining side-gating and partial top-gating provides an effective route toward compact, robust, and efficient room-temperature TeraFET detectors based on AlGaN/GaN heterostructures.

## Figures and Tables

**Table 1:** Geometry parameters of fabricated TeraFET samples.

Parameter	Sample						
	c1	d1	d2	d3	e1	e2	e3
Channel length *L* (μm)	9	9	13.5	21	9	13.5	21
Channel width *W* (μm)	3	3	3	3	3	3	3
Transition length $T_{L}$ (μm)	0	0	4	4	0	4	4
Gate separation $G_{S}$ (μm)	0	3	3	3	1	1	1
Edge gate length $G_{E}$ (μm)	0	4	8	17	3	7	16
Top gate length $G_{T}$ (μm)	5	0	0	0	1	1	1
Total gate length $G_{\Sigma }$ (μm)	5	4	8	17	4	8	17

**Table 2:** Comparison of the investigated detector geometries at 300 GHz under source–drain (SD) coupling. The responsivity values are given for the best operating points of each device.

Device	*L* (μm)	*f* (GHz)	$R_{\max}$ (V/W)	${\mathrm{NEP}}_{\min}$ (W/$\sqrt{\mathrm{Hz}}$)
FinFET	9	300	2.5	$6.4\times 10^{-8}$
EdgeFET	9	300	2.2	—
m-EdgeFET	9	300	6.5	$2.6\times 10^{-8}$
m-EdgeFET	13.5	300	1.4	$7.5\times 10^{-8}$
m-EdgeFET	21	300	3.8	$5.0\times 10^{-8}$
m-EdgeFET	9	150	5.2	$1.1\times 10^{-8}$
m-EdgeFET	13.5	150	1.5	$4.7\times 10^{-8}$
m-EdgeFET	21	150	0.5	$18\times 10^{-8}$

## Data Availability

The data that support the findings of this study are available from the corresponding authors upon reasonable request.

## Supplementary Materials

The following supporting information can be downloaded at: https://www.mdpi.com/article/10.3390/s26092701/s1, Figure S1: Calculated input impedance of the bow-tie antenna; Figure S2: THz responsivity maps of an m-EdgeFET measured without the hemispherical silicon lens. Figure S3: Noise-equivalent power for three m-EdgeFET devices with different channel lengths, and for one top-gated reference device.
