# Supplementary material for: Terahertz Antenna-Coupled Wire-Channel Field-Effect Transistors Based on AlGaN/GaN Heterostructures
Source: Sensors (Basel). 2026 Apr 27;26(9):2701. doi: 10.3390/s26092701 (PMC13165894; doi:10.3390/s26092701)
Supplement: Supplementary file 1 [file sensors-26-02701-s001.zip › sensors-4229038-supplementary.pdf]

Supplementary Materials

Terahertz Antenna-Coupled Wire-Channel Field-Effect Transistors Based on AlGaN/GaN Heterostructures

Maxim Moscotin, Justinas Jorudas, Paweł Prystawko, Miroslav Saniuk, Vitalij Kovalevskij, and Irmantas Kašalynas

This Supplementary Material provides additional data and control experiments supporting the main manuscript. Section S1 addresses the frequency-dependent antenna impedance. Section S2 provides responsivity measurements without a Si-lens to demonstrate that the reported polarization trends are not an artifact of the hemispherical silicon lens positioning. Section S3 reports the gate-voltage dependence of the noise-equivalent power for the experimental devices.

S1. Antenna impedance and frequency dependence

The antenna impedance $Z_{\text{ant}}(f)$ was obtained using full-wave electromagnetic simulations performed in CST Studio Suite 2025 (time-domain solver). Two structures were simulated:

(i) the standalone two-element bow-tie antenna with contact pads, and (ii) the full M-EdgeFET device, including two antennas, contact pads, and transistor geometry.

The metallic structures were modelled as finite-conductivity gold layers with thicknesses matching those of the fabricated devices. Excitation was implemented using a discrete port defined at the antenna feed gap, with the reference plane placed directly at the metal terminals. The complex input impedance was extracted from the simulated S_{11} parameter via

$$Z(f) = Z_0 \frac{1 + S_{11}(f)}{1 - S_{11}(f)},$$

with $Z_0 = 50\ \Omega$.

The frequency sweep ranged from 100 GHz to 1000 GHz, with sufficient frequency resolution to capture the impedance evolution and resonance features. Open (add space) boundary conditions were used to emulate free-space radiation.

S1.2 Results

Figure S1 shows the simulated complex input impedance $Z(f)$ of the standalone two-element bow-tie antenna and of the complete m-EdgeFET structure, including both antennas and device metallization. The inclusion of the transistor geometry modifies both the resistive and reactive components of the impedance due to additional distributed capacitance and channel-related reactance.

At 150 GHz, the real part of the impedance remains low ($\text{Re}(Z) \approx 25\Omega$ for both structures), while the imaginary part is predominantly capacitive. At 300 GHz, $\text{Re}(Z)$ increases to approximately 200–320 Ω, depending on whether the stand-alone antenna or the full device is considered, with a reduced reactive component compared to lower frequencies.

The comparison demonstrates that the transistor integration shifts and reshapes the impedance response, but does not introduce a narrow resonance at the frequencies of interest. Both 150 GHz and 300 GHz lie within a broadband impedance region, supporting the experimental comparison between these two frequencies and indicating that the observed responsivity trends are not dominated by an antenna resonance.

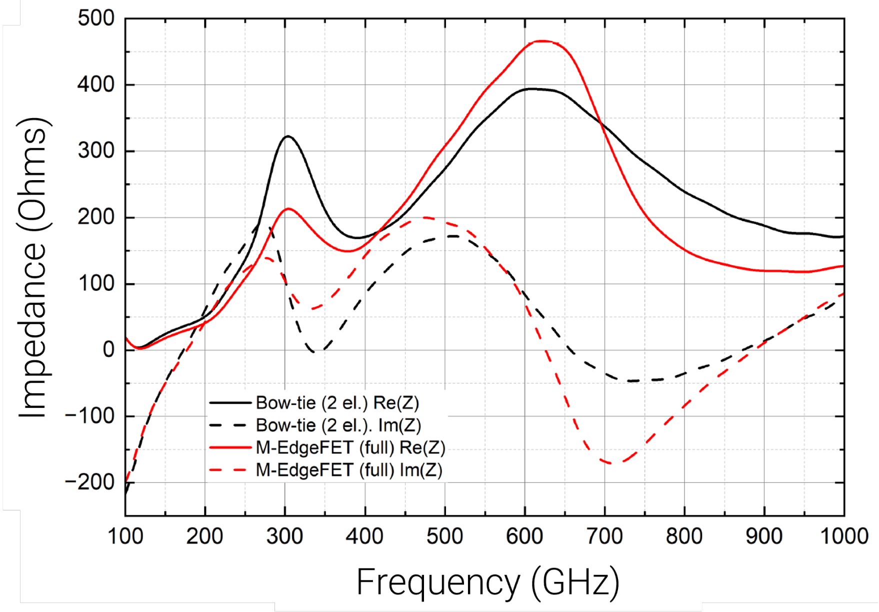

Figure S1: Calculated input impedance $Z(f)$ of the two-element bow-tie antenna (black curves) and the complete M-EdgeFET structure including antennas and device metallization (red curves). Solid lines represent the real part $\mathrm{Re}(Z)$ and dashed lines – the imaginary part $\mathrm{Im}(Z)$.

S2. THz responsivity with and without a hemispherical silicon lens

To verify that the reported polarization-dependent responsivity is not influenced by the hemispherical silicon lens, we performed control measurements with and without a Si-lens attached to the antenna-coupled FET samples, oriented selectively with respect to the focused THz beam polarization.

Figure S2 shows two-dimensional beam maps recorded without the silicon lens for (i) source–drain (SD) polarization and (ii) gate–gate (GG) polarization of the same m-EdgeFET device under otherwise identical conditions (frequency, modulation, detection chain, and gate bias) near a maximum responsivity bias point. In both cases, a well-defined focal spot is observed, demonstrating that the sample response remains polarization-dependent even in the absence of the silicon lens.

The relative amplitude and spatial localization of the response in the no-lens configuration are consistent with the trends observed in the lens-based measurements, up to an overall scaling factor associated with reduced focusing efficiency. This confirms that the polarization-dependent response reported in the main text is not an artifact of lens-induced effects.

S3. Gate-voltage dependence of NEP at 300 GHz

The noise-equivalent power (NEP) was evaluated at 300 GHz using the measured voltage responsivity together with the Johnson–Nyquist thermal noise of the channel resistance. For each gate voltage V_G, the NEP was calculated as

$$\mathrm{NEP}(V_G) = \frac{v_n(V_G)}{R_V(V_G)},$$

where $R_V(V_G)$ is the measured voltage responsivity, and the noise voltage spectral density v_n was estimated from the thermal noise expression

$$v_n = \sqrt{4k_BTR_{\mathrm{ch}}(V_G)},$$

with k_B the Boltzmann constant, T the temperature, and $R_{\mathrm{ch}}(V_G)$ the measured channel resistance.

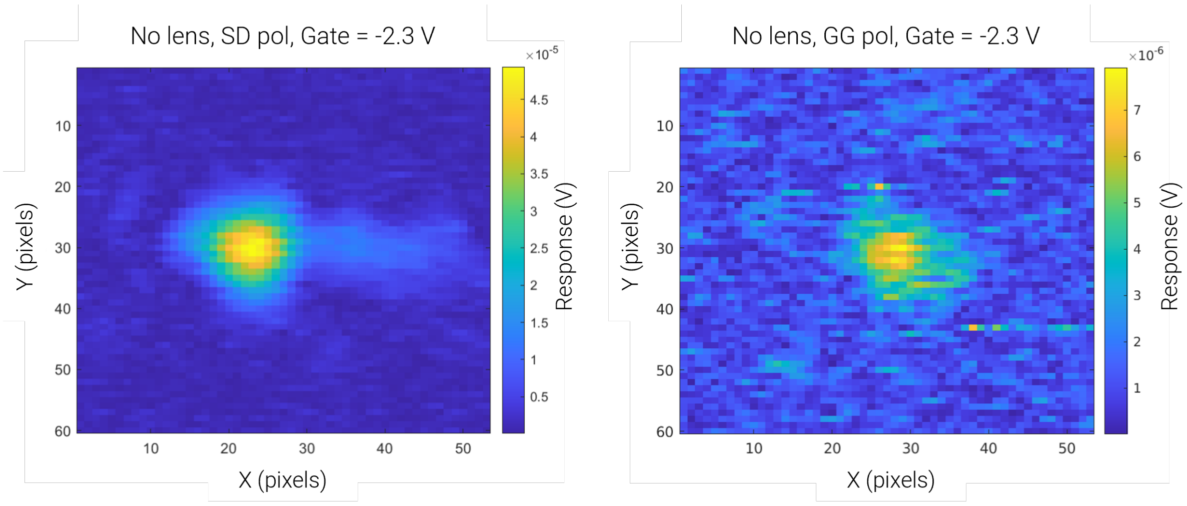

Figure S2: THz responsivity maps of an m-EdgeFET measured without the hemispherical silicon lens at $V_G = -2.3$ V. Left: source–drain (SD) polarization. Right: gate–gate (GG) polarization. The THz beam was focused directly onto the sample surface using an off-axis parabolic (OAP) mirror. A localized response maximum is observed in both polarization configurations, demonstrating that the detector retains polarization sensitivity without the silicon lens. The overall signal level is reduced compared to lens-assisted measurements due to lower optical coupling efficiency.

Figure S3 shows the resulting NEP as a function of gate voltage for four devices: three m-EdgeFETs with channel lengths $L = 21\,\mu\text{m}$, $13.5\,\mu\text{m}$, and $9\,\mu\text{m}$, and one top-gated reference device ($L = 9\,\mu\text{m}$).

All devices exhibit a pronounced minimum in NEP near the gate-voltage region where the responsivity reaches maximum. Among the measured structures, the shortest m-EdgeFET ($L = 9\,\mu\text{m}$) shows the lowest minimum NEP, reaching values on the order of $\sim 2 \times 10^{-8}$ W/$\sqrt{\text{Hz}}$. The longer-channel m-EdgeFETs exhibit slightly higher minimum NEP values, consistent with their lower responsivity. The top-gated reference device demonstrates NEP values comparable to the longer-channel m-EdgeFETs, but higher than the same-size m-EdgeFET.

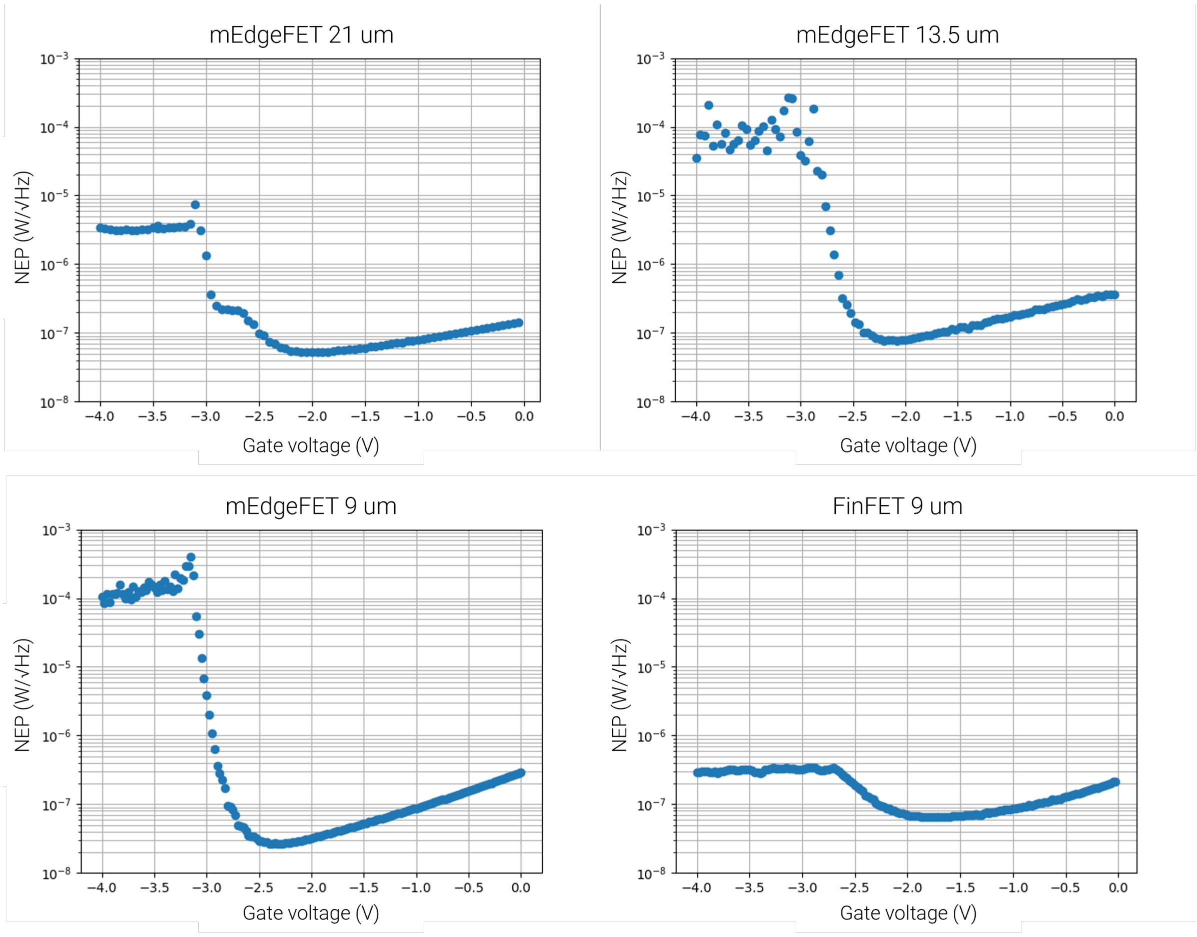

Figure S3: Noise-equivalent power NEP(V_G) at 300 GHz for three m-EdgeFET devices with channel lengths 21 µm, 13.5 µm, and 9 µm, and for one top-gated reference device (9 µm). The NEP was calculated using Johnson–Nyquist thermal noise and the measured voltage responsivity. All devices exhibit a minimum NEP near the gate bias corresponding to maximum responsivity. The shortest m-EdgeFET (9 µm) shows the lowest minimum NEP among the measured structures.
